# A Novel Operation for Ectropion

**Published:** 1855-11

**Authors:** 


A Novel Operation for Ectropion —M. Nélaton, of Paris, has lately had recourse to the following operative measures in a very bad case of ectropion. The patient has a child affected with this deformity on each side, but the left upper lid was the worst. The eyes were half open, and the cornea exposed; the free margin of the lid and the cilia were turned towards the eyebrow and the tarsal cartilage was luxuated in such a way that its superior border had become inferior. The cause of this state of things could not be learned, but it was suspected that a burn was the origin of it, as the skin was retracted and puckered. The external portion of the lid had escaped, but the destruction was complete towards the inner canthus, the free margin being only a few lines from the eyebrow.

M. Nélaton, considering that nodular retraction lasts only a certain time, judged that by stretching the parts for the space of about one year, a cicatrix might be obtained which would have lost the tentency to contraction. He therefore made a horizontal incision in the groove which separates the eyebrow from the upper lid, and the latter, having thus been freed, was brought in contact with the lower lid. The margins of the upper and lower palpebræ were then pared, for the space of about four lines external to the punctum, and kept together by three stitches. In order to reduce the luxated tarsal cartilage a strong needle was passed from below upwards through the lower lid and the cartilage, and the two parts were thus kept *in situ.* The stitches were removed three days after the operation, and the needle two days later.

The immediate results of the operation were satisfactory. The margins of the lids adhered completely on the internal half of the palpebræ fissures, the external half being open and allowing the mucous secretion to escape. The upper lid was, in this external half, noticed to ride somewhat on the lower—a circumstance which was ascribed to œdema; and as granulations were suspected on the conjunctiva, M. Nélaton cauterized with the nitrate of silver. The proceedings were completed on the 4th of February, 1854, and the patient was then freed from all constraint, and expected to spend a year at least before an effort should be made to break up the union of the lids, when retraction might no longer be apprehended, and the ectropion be completely cured. Typhoid fever, however, carried off the patient two months after the operation, hence its results could not be ascertained. We are afraid the cornea would have been found somewhat injured by the protracted immobility, nor is it at all improbable that unpleasant adhesions might have formed.—*London Lancet.*

